# Obesity, Lifestyle Behaviors, and Dietary Habits of Saudi Adolescents Living in Riyadh (ATLS-2 Project): Revisited after a Ten-Year Period

**DOI:** 10.3390/life11101078

**Published:** 2021-10-13

**Authors:** Hazzaa M. Al-Hazzaa, Nada M. Albawardi

**Affiliations:** Lifestyle and Health Research Center, Health Sciences Research Center, Princess Nourah Bint Abdulrahman University, Riyadh 11552, Saudi Arabia; namoalbwardi@pnu.edu.sa

**Keywords:** adolescents, lifestyle behaviors, obesity, physical activity, sedentary behaviors, dietary habits, sleep, health behaviors

## Abstract

Objective: Undesirable lifestyle behaviors are associated with many adverse health outcomes. This study revisited the lifestyle behaviors, dietary habits, and overweight/obesity status of Saudi adolescents from Riyadh during the year 2019/2020. We report on the rationale, design, and methodology of the current study and provide preliminary findings of the changes that occurred between the two cross-sectional studies within the span of a ten-year period. Methods: A comparison was made between two cross-sectional studies, one conducted in 2009/2010 and the other in 2019/2020, using the same design, methods, and instruments. A multistage stratified cluster random sampling technique was used to select adolescents attending public and private secondary schools. Measurements included demographics, weight, height, waist circumference, physical activity (using valid questionnaire and accelerometer), sedentary time, sleep duration, and dietary habits. Results: The total number of participants was 1262 adolescents, of which 52.4% were male, with a mean (SD) age of 16.4 ± 0.95 years. About 41% of paternal and 39.1% of maternal education levels were university degrees. Over 37% of the families earned more than SAR 20,000/month. Body mass index and waist circumference of males was larger than that of females and the overall prevalence of overweight/obesity was 40.5% with significance (*p* < 0.001) difference between males (47.3%) and females (32.8%). Between 2009/2010 and 2019/2020 datasets, there were significant changes in age (*p* < 0.001), waist circumference (*p* < 0.001), screen time (*p* < 0.001), moderate-intensity physical activity (*p* < 0.001), vigorous-intensity physical activity (*p* < 0.001), total physical activity (*p* < 0.001), and consumption of breakfast (*p* = 0.015) and fruits (*p* = 0.002). Conclusion: The most notable change between the two studies was a significant reduction in the prevalence of physical inactivity among Saudi adolescents, which was due to increased levels of moderate-intensity physical activity among Saudi females, as a result of recent positive sociopolitical changes occurring in the country over the past four years. The findings provided rich information that can be used to explore trends in overweight/obesity, lifestyle behaviors, and dietary habits among Saudi adolescents over the past ten-year period.

## 1. Introduction

According to the World Health Organization (WHO), it is estimated that 40% of the global non-communicable disease (NCD) burden appears to be linked to a few modifiable behavioral risk factors [1]. Indeed, physical inactivity and low intake of fruits and vegetables are considered by the WHO to be among the major preventable risk factors for NCDs [2]. Physical inactivity is a major risk for many NCDs and is globally responsible for 9% of premature mortality [3]. It is well recognized that lifestyle factors, such as physical inactivity, sedentary behaviors, unhealthy dietary habits, and insufficient sleep, are associated with many adverse health outcomes, including weight gain, obesity, reduced cardiorespiratory and musculoskeletal function, less favorable metabolic health, inflammation, insulin resistance and type 2 diabetes mellitus, decreased cognitive function, and negative psychological health [1,3,4,5]. On the other hand, ample evidence has shown significant associations between healthy lifestyles and increased longevity, improved health, and disease prevention [3,6,7]. Furthermore, recent research on tracking lifestyle habits shows that persistent physical inactivity from youth to adulthood is linked to an increased risk of impaired glucose metabolism in adulthood [8].

During the past few decades, Saudi Arabia experienced a major lifestyle transition accompanied by rapid economic growth and technological transformation [9,10,11]. Consequently, physical inactivity, sedentary behaviors, and consumption of calorie-dense diets and sugar-sweetened beverages increased and became prevalent among the Saudi people. This has definitely contributed to an increase in lifestyle-related NCDs in the country, including obesity, diabetes mellitus, coronary artery diseases, and hypertension [12,13,14,15,16]. Increased sedentary behaviors among adolescents [10,11] are a matter of particular concern given that emerging evidence suggests that sedentary behaviors have negative effects on health that are independent of the effects of physical inactivity [17,18,19]. In the Middle East and North Africa region, however, a recent review and meta-analysis suggests a significant increase in physical-activity prevalence among adults over the last two decades [20].

Adolescent obesity is arguably the most serious public-health challenge of recent years [21] and its prevalence among Saudi children and adolescents has been rising over the last decades [22,23]. For example, recent national surveillance shows that the prevalence of being overweight or obese among Saudi youth aged 14–24 years old was 54.1% and 51.6% for males and females, respectively [12]. Current evidence indicates that obesity is a multifactorial condition influenced by many variables both unmodifiable and modifiable, including genetic, demographic, and lifestyle variables [24]. Obesity has been shown recently to be associated with breakfast skipping [25,26,27] and insufficient sleep [28] among Saudi adolescents. Indeed, childhood obesity is associated with many amendable lifestyle factors, including sedentary behaviors, physical inactivity, and unhealthy dietary choices [29,30,31]. Therefore, monitoring the lifestyle factors that are linked with adolescent obesity is an important and meritorious research subject. It is essential to examine and monitor the reciprocal flow of lifestyle factors that influence overweight and obesity during adolescence.

At present, there is real concern about the escalating trend of unhealthy lifestyle behaviors in young people, including inactivity, sedentary habits, skipping breakfast, and increased consumption of sweetened soft drinks, as well as the possible role of these habits in the pathogenesis of childhood obesity [11,30,32]. Consequently, it is anticipated that the findings of the present research will provide valuable and updated data on the obesity status, lifestyle behaviors, and dietary habits of Saudi adolescents living in Riyadh. Continued research on the lifestyle behaviors of the Saudi youth population is of utmost importance, as diet and physical activity play important roles in maintaining health and preventing diseases [33]. In fact, the WHO has identified applied research on behavioral risk factors for NCDs as one of the key strategic approaches to be undertaken for Saudi Arabia’s Country Cooperation Strategy [34]. In addition, the Saudi Ministry of Health [35] and Saudi Vision 2030 [36] both stress the importance of a healthy lifestyle for improving the health and prosperity of the Saudi population.

In 2009, a group of Arab researchers, including those from Saudi Arabia, initiated the Arab Teens Lifestyle Study (ATLS) [11,25,26,27,28]. The main findings of the ATLS data that were collected from Saudi Arabia indicated that Saudi adolescents aged 15–19 years old had high a prevalence of being overweight or obese, as well as increased physical inactivity and sedentary behaviors, insufficient sleep, and unhealthy dietary habits [10,23,28,37,38,39]. The findings also identified several lifestyle factors associated with obesity among Saudi adolescents [25]. This follow-up cross-sectional study after ten years provides additional information on the changes that have happened since the last study conducted during the school year 2009/2010.

The current research revisited the lifestyle behaviors, dietary habits, and overweight/obesity status of Saudi adolescents attending high schools in Riyadh city during the late 2019 and early 2020 school year by using the same research design, methodologies, and instruments that were used in 2009/2010. This paper presents the rationale, methodology, and preliminary findings of the revisited ATLS study in Riyadh. Making a comparison between the two studies over the course of a ten-year period provides indirect evidence on the changing trends in obesity prevalence and lifestyle behaviors of Saudi adolescents.

## 2. Research Objectives

The objectives of the ATLS revisited research project were to:

1. Examine the prevalence of overweight, obesity, or abdominal obesity among Saudi adolescents in the current research project and compare such prevalence with the data collected ten years ago.
2. Assess the prevalence of physical inactivity, sedentary behaviors, insufficient sleep duration, breakfast skipping, and healthy/unhealthy dietary habits among Saudi adolescents relative to the prevalence rates collected during the 2009/2010 study.
3. Investigate the associations and the interactions of general obesity and abdominal obesity in Saudi adolescents with physical inactivity, sedentary behaviors, sleep duration, and dietary habits.
4. Inspect the role of sociodemographic variables on obesity, lifestyle behaviors, and the dietary habits of Saudi adolescents.
5. Assess the trends over the last ten-year period in being overweight/obese, physical activity, sedentary time, sleep duration, and the dietary habits of Saudi adolescents.

## 3. Methods and Procedures

### 3.1. Inclusion/Exclusion Criteria

All Saudi adolescents attending secondary schools (grades 10 to 12) in Riyadh city were eligible for inclusion in the study, except if they had a medical condition such as an allergy to foods, eating disorder, or a medical disorder preventing them from engaging in physical activity.

### 3.2. Ethical Considerations

Ethical approval was obtained from the Institutional Review Board (IRB) at the Princess Nourah Bint Abdulrahman University (IRB Log Number 18-0075). The research procedures were conducted according to the principles expressed in the Declaration of Helsinki. Confidentiality of the data was ensured by coding them and keeping them in restricted-access files. The participating students were not expected to be harmed in any way, be it emotional, physical, or social, from any of the procedures of the present research. The participants were informed that they could quit the study at any time if they felt uncomfortable taking part in the study or were not willing to answer the questions. Informed consent was obtained from all participating students and consent was obtained from their parents if they were younger than 18 years old. Approval for conducting this research in schools was secured from the directorate of schools at the Ministry of Education (MOE) and from the principals of the selected schools.

### 3.3. Sample Size and Sampling Technique

A representative sample was chosen using a multistage stratified cluster random sampling technique from male and female adolescents attending both public and private secondary schools in Riyadh city. The sample size was calculated using the following sample size equation for proportions: n = (z^2^pq)/d^2^), while assuming a population proportion that would yield the maximum possible sample size required (*p* = 0.50), with a confidence level of 95% and a margin of error of 5%. The sample size from each of male and female schools was 384 students. An additional 20% of participants were added to account for non-responders and missing data. Thus, the total sample size for each gender group was calculated to be 461 students, or 922 students in total. The sample was drawn in proportion to the number of students attending public and private schools, while assuming the number of students in private school to be around 30% of the total secondary students in Riyadh. The sample was drawn from the pools of secondary schools for boys and girls, based on four geographical areas plus the center of Riyadh. Schools were randomly chosen in each geographical area and classes were randomly selected from each grade of the three grades. All students in the selected classes were invited to participate in the study. Five public schools (with the average number of students = 27 × 3 classes [1 in each grade] × 5 schools = 405 male students) and three private schools (with the average number of students = 20 × 3 classes × 3 schools = 180 male students). The total number of included classes from both boys’ and girls’ schools was 48. This made the expected total number of students to be surveyed equal to 585 students from the male schools plus the same number from the female schools, for a total of 1170 boys and girls.

### 3.4. Measurements

#### 3.4.1. Demographics and Socioeconomic Status (SES)

These measures included chronological age, gender, maternal and paternal education, and family income. Parental education levels encompassed intermediate or less, high school, university, and post-graduate. Family income in Saudi Riyal (SAR) included the following categories: SAR 10,000 or less, SAR 10,001–SAR 20,000, SAR 20,001–SAR 30,000, and more than SAR 30,000 (USD 1 = SAR 3.75).

#### 3.4.2. Anthropometric Measurements

Anthropometric variables included the body weight and height of the adolescents, as well as calculated body mass index (BMI). Measurements were performed in the morning by a trained researcher according to standardized procedures. Body weight was measured to the nearest 100 g using calibrated portable medical scales (Seca 770 model scale, Seca, Hamburg, Germany). All measurements were performed with minimal clothing and without shoes. Height was measured to the nearest centimeter using a calibrated measuring rod while the subject was in a full standing position without shoes. BMI was calculated as the ratio of weight in kilograms to height in meters squared.

The International Obesity Task Force (IOTF) age- and sex-specific BMI cut-off reference standards [40] were used to identify overweight and obesity among adolescents between the ages of 14 and 17 years. For participants 18 years and older, we used the WHO adult cut-off points of 25–29.9 kg/m^2^ to define overweight and 30 kg/m^2^ and higher to define obesity. Waist circumference (WC) was measured horizontally at navel level at the end of a gentle expiration to the nearest 0.1 cm using a non-stretchable measuring tape. Waist-to-height ratio (WHtR) was calculated as the ratio between WC in cm and height in cm. A WHtR cut-off point of 0.50 was used to define abdominal obesity in both males and females [41,42]. In addition, we calculated body shape index as WC/((BMI ^2/3^) × (Height ^1/2^)).

#### 3.4.3. Physical Activity

The Arab Teen Lifestyle Study (ATLS) questionnaire was used to collect lifestyle variables [10,37]. The questionnaire was previously shown to be valid and reliable for assessing physical activity and other lifestyle habits in youth from 14 to 25 years of age [10,37,43,44]. The questionnaire collected information on the frequency, duration, and intensity of light-, moderate-, and vigorous-intensity physical activities during a typical week. The questionnaire covered several domains of activity including transport, household, fitness, and sporting and leisure-time activities. The total time in minutes spent on all activities per week, as well as time spent in moderate- and vigorous-intensity physical activities, were used as indicators of physical-activity levels.

To determine the participants’ levels of physical activity, we used the total activity energy expenditure in Metabolic Equivalent in minutes per week (METs-min/week) and the METs-min/week spent in each of the moderate- and vigorous-intensity physical activities. This measurement is based on MET values corresponding to physical-activity intensity, according to the adults’ and youths’ compendium of physical activities [45,46]. To calculate the percentage of adolescents who met the daily-physical-activity recommendations, we used two cut-off scores equivalent to 1 h per day of moderate-intensity (4 METs) physical activity or 1 h per day of moderate- to vigorous- intensity (6 METs) physical activity. We then converted these two amounts of exercise into METs-min per week, corresponding to 1680 METs-min/week (60 min per day × 7 days per week × 4 METs) and 2520 METs-min per week (60 min per day × 7 days per week × 6 METs).

In addition to the ATLS questionnaire, an accelerometer (GENEActiv from Activinsights Ltd., Cambridgeshire, UK) was used to assess participant levels of physical activity by objective measure. An accelerometer was placed on the non-dominant wrist of each adolescent continuously for seven days of free-living. Data were taken even while sleeping and bathing. The GENEActiv accelerometer is a lightweight and water-resistant device. Clear instructions were given to the adolescents to ensure their compliance during the entire seven-day period.

#### 3.4.4. Sedentary Behaviors

Questions on sedentary behaviors were part of the ATLS questionnaire. They were intended to determine important information from the participants about the typical daily time spent on all types of screen time, including time spent viewing TV, playing video games, and computer and internet recreational use. Participants were asked to provide the average number of daily hours during both weekdays and weekends that were spent on screen time. For the sedentary time cut-off hours, we used the American Academy of Pediatrics guidelines of a maximum of 2 h per day [47]. Due to the high percentage of screen time exhibited by students, we also used a cut-off of below or above 3 h of screen time. In addition to the ATLS questionnaire, the accelerometer could record sedentary time as well.

#### 3.4.5. Sleep Duration

Sleep duration during both weekdays (school days) and weekends was assessed through the questionnaire. We defined insufficient sleep (short sleepers) as sleeping fewer than 8 h per night, according to the definition of the National Sleep Foundation for school-age adolescents 14–17 years [48]. In addition, accelerometers were used to record sleep duration.

#### 3.4.6. Dietary Habits

The ATLS questionnaires included ten specific questions related to intake frequency of certain dietary habits during a typical (usual) week. The questions asked the participants to state how many times per week they consumed breakfast, vegetables (cooked and uncooked), fruits, milk and dairy products, sugar-sweetened drinks (including soft drinks), fast foods, donuts/cakes, sweets and chocolates, and energy drinks. The questions covered both healthy and unhealthy dietary habits. The students were given a choice of answers, ranging from zero intake (never) to a maximum intake of seven days per week (every day).

#### 3.4.7. Training Research Assistants

The principal and co-principal investigators selected a group of research assistants and a group of volunteers and trained them on how to properly conduct the research measurements, as well as how to handle any circumstances that might occur during the data collection in schools. They were also provided with the school names, locations, phone numbers, and letters of approval from the MOE. In addition to the investigators, two of the senior staff of the Lifestyle and Health Research Center (LHRC) were assigned to closely supervise the research team and the data collection.

#### 3.4.8. Research Timeline

The data collection started at the beginning of October 2019 and finished at the beginning of March 2020. Data collection ceased when there were periodic exam days or bad weather conditions, as these two factors could interfere with normal levels of physical activity or sedentary behaviors. The collection finished just before any restriction (shutdown) was imposed on schools due to COVID-19.

### 3.5. Data and Statistical Analyses

Data were entered into an SPSS data file, checked, cleaned, and analyzed using SPSS Statistics 22 (IBM, Chicago, IL, USA). To avoid over-reporting, physical-activity scores were cleaned and truncated at reasonable and realistic levels [10,37], taking into account the fairly long time spent in learning at schools, doing homework, sleeping, and TV time. The maximum total time spent on physical activity per week was truncated at 28 h or 4 h of maximal physical activity per day [10,37]. In addition, the maximum number of stair levels taken by students per day was capped at 30 floors. The combined time of TV viewing, computer use, and Internet time was truncated at 16 h per day [10,37].

Continuous variables were tested for normality by different methods. The normally distributed variables were summarized using frequencies, means, standard deviations, or standard errors. In cases of severe non-normal distribution, we applied the equivalent appropriate non-parametric tests of significance and reported median and interquartile-range values. Differences between breakfast skippers and non-skippers, boys and girls, public versus private schools, active and inactive participants, sufficient versus insufficient sleepers, and participants with overweight/obesity and without overweight/obesity were compared using the classical statistical tests of significance (*t*-tests, ANOVA, and 2- and 3-way MANCOVA). To study the quantitative effects of different independent variables on youth adiposity, and on dependent outcome variables such as sedentary behaviors, sleep duration, demographics, and socio-economic status, we used logistic regression and other multivariable statistical methods, while controlling for confounders such as age, gender, and sociodemographic variables. Pearson’s correlation and linear regression were used when appropriate, and the level of significance was set at a value of ≤0.05.

## 4. Preliminary Results

### 4.1. Demographics and SES

We collected data from a total of 1262 participants, of which 52.4% were male. The response rate was extremely high, reaching 99.2%. The mean age for the participating adolescents was 16.4 ± 0.95 years. About 41% of paternal and 39.1% of maternal education levels were university degrees. Over 37% of the students’ families earned more than SAR 20,000 Saudi Riyals per month. Table 1 presents the number of students selected from the 15 participating schools (48 classes in total).

Table 2 shows the number of participants, their ages, and the proportion of adolescents from public and private schools relative to gender. Nearly 75% of adolescents came from public schools in Riyadh, with more girls (29.5%) than boys (21.1%) selected from private schools.

### 4.2. Anthropometric Measurements

The anthropometric measurements of the Saudi adolescents are displayed in Table 3. As expected, there were significant (*p* < 0.001) differences between boys’ and girls’ weights and heights. Further, the BMI of boys was higher than that of girls. The prevalence of overweight was similar between boys (20.8%) and girls (20.4%). However, obesity prevalence was significantly (*p* < 0.001) higher in boys (26.4%) than in girls (12.4%).

### 4.3. Proportional Changes between 2009/2010 and 2019/2020 Data

**Figure 1:**
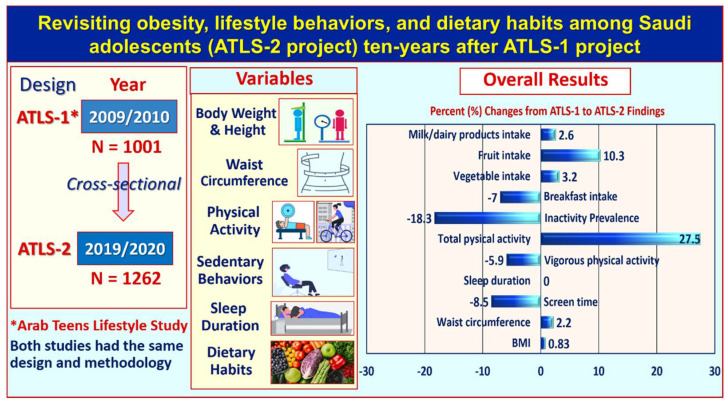
Graphical illustration of the design and overall findings of the studies conducted in 2009/2010 and again in 2019/2020.

Table 4 shows the proportional changes that occurred between selected anthropometric and lifestyle variables between the 2009/2010 and 2019/2020 datasets. There were significant changes in chronological age (*p* < 0.001), waist circumference (*p* < 0.001), average screen time (*p* < 0.001), moderate-intensity physical activity (*p* < 0.001), vigorous-intensity physical activity (*p* < 0.001), total physical activity (*p* < 0.001), and consumption of breakfast (*p* = 0.015) and fruits (*p* = 0.002). Figure 1 also illustrates the design and the overall changes between the Arab Teens Lifestyle Study (ATLS) 1 and 2.

## 5. Discussion

The aim of the present study was to revisit the lifestyle behaviors, dietary habits, and overweight/obesity status of Saudi adolescents attending high schools in Riyadh city during the school year 2019/2020, by using the same research design, methodologies, and instruments that were used to collect lifestyle data from Saudi adolescents in 2009/2010. In this article, we have presented the rationale, methodology, and preliminary findings of the ATLS-2 study, which was conducted recently in Riyadh. The main findings indicate that there have been both some positive and some negative changes in anthropometric measurements, lifestyle behaviors, and dietary habits over the course of the past ten-year period.

This is the first report to describe and discuss the trends in lifestyle behaviors in Saudi adolescents. Continuous tracking of lifestyle behaviors is crucial in order to identify risk-groups with low physical activity, increased sedentary time, insufficient sleep, and unfavorable dietary habits. It is also important to promote healthy behaviors and guide interventions. It has been well acknowledged that continued research on the lifestyle behaviors of Saudi youth is of utmost importance as diet, sleep, sedentary behaviors, and physical activity play important roles in maintaining health and preventing diseases [33]. In fact, the WHO has identified applied research on behavioral risk factors for NCDs as one of the key strategic approaches to be undertaken for Saudi Arabia’s Country Cooperation Strategy [34]. In addition, the Saudi Ministry of Health [35] and Saudi Vision 2030 [36] stress the importance of a healthy lifestyle for improving the health and prosperity of the Saudi population.

The present study provides brief yet important information related to trends occurring in overweight/obesity and lifestyle behaviors among Saudi adolescents over the last ten-year period. The Kingdom of Saudi Arabia (KSA) has a population exceeding 34 million, and 24% of its population is adolescents aged 15–19 years [49]. The ATLS-2 project targeted lifestyle habits of adolescents at the secondary school level, which typically spans an age range between 14 and 19 years. These formative years of adolescence signify a crucial stage in the human life cycle, where lifestyle habits are formed and established. During this period, adolescents become more independent and have increased access to food choices apart from those available at home. It is also during this period that the adolescent increases his/her social interaction with peers of a similar age and develops individual eating habits and physical-activity patterns. Research has shown that dietary habits are established in the mid-teens and are closely associated with lifestyle [50]. A better understanding of the relationships of healthy behavior among youth is necessary for effective prevention and management of lifestyle-related risk factors.

The WHO recently released a milestone document emphasizing the importance of increasing physical activity and decreasing sedentary time for all ages, including adolescents [5]. The WHO also included nutrition as one of the important components of the Global Strategy for Women’s, Children’s and Adolescents’ Health (2016–2030). Their report emphasizes that global malnutrition is changing from stunting and nutritional anemia to excess body weight. The WHO identified that institutions and policies are essential components for promoting a good nutritional status for countries [51].

Earlier research from the ATLS project conducted during the year 2009/2010 showed that 45.5% of young Saudi males and 78.1% of young Saudi females were inactive [10]. In addition, 84% of males and over 91% of females had spent more than 2 h per day on screen time [10]. It was clear from the findings that females appeared consistently at a higher risk of both physical inactivity and sedentary behaviors than males [10]. There were also significant differences by gender in physical activity and screen time [10]. It is now well recognized that sedentary behaviors are associated with adverse health outcomes in a way that appears to be different from those attributed to a lack of physical activity [52]. Past findings pertaining to Saudi adolescents showed contrasting results relative to gender and school type. Saudi males in public schools were more active than in private schools, whereas the opposite was true for females [10,53]. This may have changed in the present time, as public schools recently have begun introducing physical education programs in girls’ schools. Compared with females, male adolescents in private schools had higher odds of being overweight or obese than those in public schools [25]. Moreover, compared with females, Saudi males spent significantly more time per week in leisure-time physical activities but not in non-leisure-time physical activity [54]. In addition, there was a significant interaction between genders by obesity level in leisure-time physical activity. Gender, and other factors, predicted total duration spent in leisure-time and non-leisure-time physical activity [54].

The earlier ATLS study on Saudi adolescents indicated that unhealthy dietary habits were widely prevalent among them, including skipping breakfast, low intake of fruit and vegetables, and high consumption of fast foods, French fries/potato chips, and chocolates and candy [10]. The previous ATLS study also showed that unhealthy behaviors, such as increased screen time and unhealthy dietary habits, appeared to aggregate among Saudi adolescents. Healthy dietary habits (intake of breakfast, fruit, vegetables, and milk/dairy products) associated mostly with increased levels of physical activity, whereas unhealthy dietary habits (higher consumption of sugar-sweetened drinks, fast foods, cake/donuts, and energy drinks) were related most to higher screen time. [38]. We also observed in previous ATLS research a high prevalence of short sleep duration among Saudi adolescents [28].

The preliminary findings of the current study found a substantial increase in total activity energy expenditure among Saudi adolescents compared with similar findings from the study conducted a decade ago. The increase was more noticeable in moderate-intensity physical activity, especially among females. Such increase in physical activity among Saudi females can be attributed to the recent socio-political changes in the country that allowed more opportunities for Saudi females to be physically active [35,36]. These positive socio-political changes have provided more autonomy and opportunities for Saudi women to participate in many aspects of societal life. The country introduced physical education curricula and programs for girls in all schools in 2017, permitted women to drive and travel more independently in 2018, and granted licenses to open private gyms for women, all of which have allowed for more prospects for women to engage in sporting and physical activities.

Longitudinal studies among adolescents have revealed that higher moderate- to vigorous-intensity physical activity is positively associated with favorable bone health, cardio-metabolic health and fitness, blood lipid profile, blood pressure, insulin sensitivity, and reduced obesity [55,56,57,58,59,60]. Physical activity also plays an important role in the prevention of overweight and obesity in childhood and adolescence [61]. A review of studies employing mostly self-reports of physical activity found mixed results and identified inconsistent magnitudes of change when looking for trends in different contexts of physical activity for children and adolescents over the last few decades [62]. The review also found consistent declines in active transport, particularly cycling [62]. In the present analysis of Saudi data, cycling contributed minimally to total physical activity among Saudi adolescents in both studies conducted in 2009/2010 and 2019/2020. Overall physical activity remained stable among German youths between 2003 and 2017 [63]. However, the study observed an increase from baseline (2003 to 2006) to Wave 2 (2014 to 2017) in physical activity participation from 53.5% to 60.0% in sports clubs [63]. Other studies demonstrated a decline in physical activity among adolescents. An Australian study observed context-specific declines in children’s physical activity participation over a period from 1985 to 2013. Early adolescent females experienced the most declines in most contexts, which included school active transport, organized sport, physical education, and school break-times [64]. In addition, the proportion of Iranian adolescents who displayed low levels of physical activity increased from 2006 to 2011 in both urban and rural areas [65]. The results of a recent systematic review reporting longitudinal changes in moderate- to vigorous- intensity physical activity (MVPA) among children and adolescents showed that the relative change in MVPA per year pointed to a decline of −3.4% (95% CI, −5.9 to −0.9) in boys and −5.3% (95% CI, −7.6 to −3.1) in girls, across all age groups [66].

The current study showed that screen time among Saudi adolescents decreased by 8.5% over the ten-year period. The World Health Organization (WHO) recommends reducing sedentary behaviors across all age groups and abilities [5]. A high sedentary time among children and adolescents was found to relate to adiposity [19]. Using accelerometers, participants in the Gateshead Millennium Study were assessed for sedentary time at age 7, 9, 12, and 15 years. The findings showed that increased sedentary time from 7 to 15 years was associated with increased adiposity [19]. Additionally, participants in the Gateshead Millennium Study cohort showed an increase in sedentary time to almost 75% of waking hours at age 15 years [67]. Further, continuous testing of a large, United States population showed that the prevalence of sitting while viewing screens at least 2 h per day was high and the estimated total sitting time increased between 2007 and 2016 from 7.0 to 8.2 h per day among adolescents [68]. Temporal trends in screen viewing use among adolescents across 30 countries were examined between 2002 and 2010 [69]. There was a slight decrease in sedentary time in most of the 30 countries among both boys and girls. Such decrease was more than balanced by a sharp increase in computer use across all countries. The study called for examining the determinants of sedentary behaviors among adolescents in those participating countries [69]. With increased reliance on computers and the internet, young people may substitute physical activity for sedentary time. A recent Norwegian study tracked physical activity from 2005/2006 to 2011/2012 using accelerometers and found that both children and adolescents substituted time spent in light physical activity for time spent sedentary [70].

Our current study exhibited modest changes in BMI among Saudi adolescents over the ten-year span. This finding is corroborated by data from the findings of the Non-communicable Disease Risk Factor Collaboration (NCD-RisC) study, which included 65 million school-aged children and adolescents in 200 countries including Saudi Arabia, and estimated trends from 1985 to 2019 in mean height and mean BMI [71]. The results of the NCD-RisC study showed that 19-year-old girls in some countries including Saudi Arabia grew much taller, however, their BMI increased about the same as the global median [71]. A recent study involving Norwegian adolescents reported that moderate to vigorous intensity physical activity in the first year of secondary school was not associated with changes in body composition measures after two-years’ follow-up [72]. However, results of the International Children’s Accelerometry Database (ICAD) showed that overweight or obesity was related to physical inactivity [73].

Our study showed that breakfast intake was reduced by 7%. Similar studies have reported reductions in breakfast consumption over the years. For instance, the three-day dietary records of children and adolescents aged 2–18 years collected between 1986 and 2007 in the Dortmund Nutritional and Anthropometric Longitudinally Designed Study demonstrated a significant reduction in regular breakfast intake in 6 to 12- and 13 to 18-year-olds [74]. Fruit and vegetable intake by Saudi adolescents over a ten-year period have increased by 3.2% and 10.3%, respectively. Analyses of Danish data from the international Health Behaviour in School-aged Children (HBSC) study collected in 1988, 1991, 1994, 1998, 2002, and 2006 revealed a significant decrease in prevalence of students eating fruit at least once daily from 1988 to 2002. However, in all age and gender groups, a significant increase in fruit consumption occurred between 2002 and 2006 [75]. On the other hand, dietary data from the NHANES 1988–1994, 1999–2002, and 2003–2008 showed that changes in dietary behaviors over the past two decades were modest and were not accompanied by a significant increase in energy intake. [76]. Using data from the Youth Risk Behavior Surveillance System between 2005 and 2017 to assess the change in disparities in meeting guidelines for fruit and vegetable intake, it was found that sex-specific and age-specific disparities increased for meeting fruit and vegetable intake [77]. In addition, samples from Australian secondary school students aged 12–17 years were surveyed in 2009/2010 and 2012/2013. The results showed that females had significantly greater odds of failing to meet guidelines for vegetable intake than males [78]. Our current study showed a trend toward a small increase of 2.6% in milk/dairy-product intake over time. However, a study conducted on American high school students during 2007–2011 showed that daily milk consumption did not significantly change from 2007 to 2011, but during 2011 to 2015 daily milk consumption decreased from 44.3% to 37.4% [79].

Some of the pressing questions that might be answered by the current ATLS-2 findings include the following:

- What is the prevalence of obesity and abdominal obesity among Saudi adolescents? Have these numbers decreased or increased compared to similar measurements collected ten years ago?
- What is the prevalence of physical inactivity and sedentary time among Saudi adolescents, and how has this changed in the past ten years?
- What is the prevalence of insufficient sleep duration among Saudi adolescents, and how has this decreased or increased compared to data collected in the year 2009/2010?
- What is the prevalence of breakfast skipping among Saudi adolescents, and how has this decreased or increased compared to the prevalence in the year 2009/2010?
- What is the prevalence of vegetable, fruit, and milk/dairy-product intake among Saudi adolescents, and has this decreased or increased since the first study?
- What is the prevalence of unhealthy dietary habits, such as consumption of fast food, French fries, or sugar-sweetened drinks among Saudi adolescents, and has the prevalence of this consumption decreased or increased since the year 2009/2010?
- What is the correlation between the physical inactivity and sedentary time findings obtained by the ATLS questionnaires and the data obtained by accelerometers?
- What are the major lifestyle behaviors or dietary habits that may be influencing obesity, abdominal obesity, or breakfast skipping among Saudi adolescents?
- Are there any gender differences relative to obesity, lifestyle behaviors, or eating habits among Saudi adolescents?
- What are the associations between overweight and obesity status and breakfast intake and other behaviors among Saudi adolescents?
- What are the relationships between demographics and SES with lifestyle behaviors or eating habits among Saudi adolescents?
- Finally, is there a clustering of health-related behaviors among Saudi adolescents? A clustering of unhealthy lifestyle habits such as unhealthy dietary practice, inactivity, and sedentary behaviors may be a major public-health concern.

### Strengths and Limitations

The current research has many strengths and some limitations. Among the strengths of the present study is that this research collected a large volume of information related to many variables of interest, which provides a bigger picture of exposures and outcomes. It collected comprehensive and rich data related to general obesity, abdominal obesity, demographics and SES, physical activity/inactivity, screen time, and dietary habits. Another strength of the current research is the use of a combination of a validated self-reported questionnaire and an objective measure (the accelerometer) for collecting data on physical activity, sedentary behavior, and sleep.

As to the limitations of the study, the adolescents’ dietary behaviors were self-reported. However, the instrument has been used extensively in the past in adolescents and youths and has proven to be useful and reliable [10,38,80]. In addition, accelerometer measurement reactivity may influence measurement of physical activity and sedentary behaviors [81]. Nevertheless, we continually collected seven-day accelerometer data, which could reveal if such phenomena existed. Finally, the cross-sectional design, which cannot imply any causality, is a potential limitation of the current study. However, there are still significant contributions that can be made by cross-sectional studies, and they can pave the road to more prospective studies.

## 6. Conclusions

We collected an enormous amount of anthropometric and lifestyle-variable data from Saudi adolescents during this research project and compared the findings with those obtained from a similar study conducted in 2009/2010. There were several parameters that changed over the ten-year period. Most notable was the significant reduction in the prevalence of physical inactivity among Saudi adolescents, which was due to increased levels of moderate-intensity physical activity among young Saudi females. The study provides rich information that can be used to explore trends in overweight/obesity concerns, lifestyle behaviors, and dietary habits among Saudi adolescents over the period from 2009/2010 to 2019/2020. It is clear that many of the favorable lifestyle changes were due to significant improvement in females’ lifestyle behaviors, including physical activity and increased consumption of fruit and milk/dairy products. The current study has numerous implications for Saudi adolescent health and well-being. It is anticipated that the research findings will provide updated information on obesity prevalence in adolescents living in Riyadh. The findings will also provide more understanding of the lifestyle-related determinants and associations of obesity among Saudi adolescents. This study will have significant and far-reaching implications for effectively planning and executing preventive and promotional programs geared toward the health and prosperity of Saudi adolescents, especially those with excess weight problems.

## Figures and Tables

**Table 1:** The selected schools and number of students relative to gender and type of school.

SN	School Name	Gender	Type of School	Number of Participants
1	First High School	Females	Public	85
2	High School 39	Females	Public	73
3	High School 58	Females	Public	97
4	High School 68	Females	Public	73
5	High School 139	Females	Public	96
6	Tarbiah Namozhejiah High School	Females	Private	63
7	Sorooq Almarifah High School	Females	Private	54
8	Near Private High School	Females	Private	60
9	Imam Shawkani High School	Males	Public	105
10	Prince Sultan Complex High School	Males	Public	89
11	Muhammed bin Wasie High School	Males	Public	115
12	Abeer High School	Males	Private	66
13	Fursan High School	Males	Private	74
14	Maahad Asameh High School	Males	Public	113
15	Shoura High School	Males	Public	99
Total selected students = 1262 (Females = 601; Males = 661)				

**Table 2:** Cross-tabulation of the demographic factors of the participants relative to gender and school type (*n* = 1262).

Variable	All	Males	Females	*p*-Value *
Number of participants	1262	661 (52.4%)	601 (47.6%)	-
Age (year)	16.4 ± 0.95	16.4 ± 0.94	16.3 ± 0.96	0.050
School type (%)				0.001
Public	74.8	78.8	70.5	
Private	25.2	21.2	29.5	

* Chi-square tests for the proportion.

**Table 3:** Anthropometric characteristics of the participants relative to gender.

Variable	All *n* = 1261	Male *n* = 660	Female *n* = 601	*p*-Value *
Body weight (kg)	65.6 ± 20.9	73.2 ± 23.1	57.3 ± 14.3	<0.001
Body height (cm)	163.6 ± 8.9	169.7 ± 6.8	157.0 ± 5.5	<0.001
Body mass index (kg/m^2^)	24.3 ± 6.6	25.3 ± 7.4	23.2 ± 5.4	<0.001
Overweight + obesity **	40.5	47.3	32.8	<0.001

Data are means ± standard deviations or percentage. * *t*-test for independent samples or Chi-square tests for the proportion for the differences between males and females. ** Based on IOTF cut-off values (reference number 40).

**Table 4:** Mean proportional changes (%) between 2009/2010 and 2019/2020 findings. * Chi-square tests for the proportion.

Variable	Change	Percent Change (%)	*p*-Value *
Age (year)	Increased	2.5	**<0.001**
Body weight (kg)	Increased	3.1	0.107
Body mass index (kg/m^2^)	Increased	0.83	0.582
Waist circumference (cm)	Increased	2.2	**0.027**
Screen time (hours/day)	Decreased	8.5	**<0.001**
Sleep duration (hours/night)	No change	0.0	0.558
All moderate-intensity physical activity (METs-min/week)	Increased	111.9	**<0.001**
All vigorous-intensity physical activity (METs-min/week)	Decrease	5.9	**<0.001**
Total physical activity (METs-min/week)	Increased	27.5	**<0.001**
Inactivity prevalence (<1680 METs-min/week)	Decreased	18.3	**<0.001**
Breakfast intake (day/week)	Decreased	7.0	**0.015**
Vegetable intake (day/week)	Increased	3.2	0.247
Fruit intake (day/week)	Increased	10.3	**0.002**
Milk/dairy-product intake (day/week)	Increased	2.6	0.187

## Data Availability

All data generated or analyzed during this paper are included in this published article. Any additional data will be available from the corresponding author upon reasonable request.
